# The speed of information propagation in the scientific network distorts biomedical research

**DOI:** 10.7717/peerj.12764

**Published:** 2022-01-10

**Authors:** Raul Rodriguez-Esteban

**Affiliations:** Roche Pharmaceutical Research and Early Development, Roche Innovation Center Basel, Basel, Switzerland

**Keywords:** Scientometrics, Information science, Knowledge management, Citation network, Knowledge diffusion

## Abstract

Delays in the propagation of scientific discoveries across scientific communities have been an oft-maligned feature of scientific research for introducing a bias towards knowledge that is produced within a scientist’s closest community. The vastness of the scientific literature has been commonly blamed for this phenomenon, despite recent improvements in information retrieval and text mining. Its actual negative impact on scientific progress, however, has never been quantified. This analysis attempts to do so by exploring its effects on biomedical discovery, particularly in the discovery of relations between diseases, genes and chemical compounds. Results indicate that the probability that two scientific facts will enable the discovery of a new fact depends on how far apart these two facts were originally within the scientific landscape. In particular, the probability decreases exponentially with the citation distance. Thus, the direction of scientific progress is distorted based on the location in which each scientific fact is published, representing a path-dependent bias in which originally closely-located discoveries drive the sequence of future discoveries. To counter this bias, scientists should open the scope of their scientific work with modern information retrieval and extraction approaches.

## Introduction

The wide communication of scientific discoveries across the scientific community is an essential element of scientific research. Delays in the propagation of scientific discoveries, particularly referring to the existence of scientific silos, have long been bemoaned for hindering this process (*e.g.*, Leischow et al., 2008; Vodovotz & An, 2013; Törmä, 2019) by introducing a bias towards knowledge that is produced within a scientist’s closest community. Analogous to corporate knowledge silos, there are at least three aspects that would define scientific silos: (1) enormous growth in the knowledge available to scientists, (2) organization of scientists into communities and (3) slowing of the propagation of scientific knowledge between those communities. Regarding the first aspect, the growth of information available for scientific research (Larsen & von Ins, 2010; Bornmann & Mutz, 2015) represents a challenge for individual scientists as information seekers (Landhuis, 2016) and producers (Genova, Astudillo & Fraga, 2016). In a perfect world, scientists would possess complete knowledge of all existing scientific information and select their research goals accordingly. Abundance of information, however, can represent its own “resource course” challenge. One could paraphrase the famous corporate knowledge-management adage (Sieloff, 1999) by saying: “if only science knew what science knows.” In this respect, the field of literature-based discovery (LBD) has propounded the existence of “undiscovered public knowledge” concerning facts that have never been put together before because of the disparate venues in which they were published (Swanson, 1986; Bekhuis, 2006; Thilakaratne, Falkner & Atapattu, 2019). Thus, there is a recognition that the milieu in which a discovery is published influences its later use by the scientific community due to the sheer abundance of existing scientific knowledge. This phenomenon can appear even within scientific fields that grow larger, with new publications struggling to garner attention (Chu & Evans, 2021).

With respect to the second aspect, it has been shown that scientific publications are anchored around communities of scientists (Bruggeman, Traag & Uitermark, 2012; Shia, Foster & Evans, 2015; Fortunato et al., 2018), which go beyond traditional scientific communities (*e.g.*, university departments, scientific organizations), representing a self-organizing process. This process might be encouraged by an institutional bias against interdisciplinary research (Bromham, Dinnage & Hua, 2016; Baumwol et al., 2011), which would hamper collaboration across communities, despite recent trends towards fostering interdisciplinary research in systems and translational sciences (Luke et al., 2015; Auffray, Chen & Hood, 2009). It could also be a consequence of human cognitive limitations, due to scientists’ bounded capacity to learn and produce new knowledge and as a response to an increasingly more complex scientific landscape (Rodriguez-Esteban & Loging, 2013).

The third aspect, and the focus of this study, relates to the speed in which information propagates across the scientific network, and which ultimately has an impact on the direction of scientific progress. The existence of inefficiencies in the propagation of scientific information across the scientific network would increase the likelihood of certain discoveries to be based on facts published within closer communities, such as a silo, to the detriment of discoveries based on facts coming from more separated communities. Because new discoveries feed on past discoveries in a path-dependent manner (Soler, Trizio & Pickering, 2015; Tambolo, 2017; Heimeriks & Boschma, 2014), this dynamic could affect the long-term outcome of scientific research.

While siloization, and solutions that try to address it, have been a recurrent topic of scientific debate, no effort has been made to-date to quantify its negative impact on scientific progress, particularly its effect on the slowdown in the propagation of scientific facts, leading to the delay of certain discoveries and to the acceleration of others. This first attempt focuses on measuring the propagation of scientific facts about relations between compounds, genes and diseases. While the propagation of other scientific facts would be of interest, compounds, genes and diseases are more amenable to the analysis pursued in this study due to the abundance of relevant curated data. Moreover, they are of broad interest in biomedical discovery, including clinical, pharmaceutical and translational research. In pharmaceutical research, for instance, the dynamics of propagation of new drug-related knowledge across diseases has been seen to relate to measures of disease similarity (Rodriguez-Esteban, 2016). Hence, disease similarity measures could be associated to the speed of knowledge propagation and affect the likelihood by which compound-gene-disease associations are made in the pursuit of new medicines.

Because defining distance within the scientific landscape is challenging, a surrogate distance measure—the citation distance—is used in this study to represent the separation between publications. The citation distance, also known as geodesic distance or shortest-path distance, is here the topological distance between publications in the citation network and has been used to measure the relatedness of scientific communities (Shibata, Kajikawa & Sakata, 2011) and journals (Franceschet, 2012), or to measure interdisciplinarity (Silva et al., 2013).

Results of the analysis show that the citation distance between two published facts influences the probability that they will lead to a new discovery and thus signal the importance that the large-scale structure of relations between scientific publications have in distorting scientific progress.

## Materials & Methods

Scientific discovery can be modeled as a process in which facts are progressively connected to each other, thereby building growing networks in which the discovery of new facts is connected to already discovered facts (Cokol et al., 2005; Rzhetsky et al., 2015). The scientific discovery model employed in this study is inspired by the ABC model used in literature-based discovery (LBD) (Smalheiser, 2012; Thilakaratne, Falkner & Atapattu, 2019) and it is based on undirected networks of up to 3 nodes (A, B and C). The nodes are particular elements that are the focus of research and the edges are relations between those elements that have been published in scientific publications. These networks are built sequentially over time: the edge AB is associated to the relation that is published first, the edge BC is associated to the second one, and the edge AC to the third one. Based on the time sequence order, the nodes are labeled appropriately as A, B or C. At any given point in time, and based on the existing published literature, there are networks with 1, 2 and 3 edges. For networks with all 3 edges, we say that AB and BC *enabled* the discovery of AC, even if there is no direct evidence of that, by virtue of precedence. AB and BC are considered “enabling facts” and AC, a “new discovery.” Networks with 2 edges comprise *potentially* enabling pairs of facts (*i.e.,* AB and BC), which could enable a new discovery AC in the future.

In a full, three-edge ABC network, the time elapsed for a new discovery is the time between the publication of BC and the publication of AC. In a two-edge network (*i.e.,* AC does not exist), the time elapsed is measured between publication of BC and the cut-off time (January 1, 2020; which is based on data availability and download dates when this study was performed). This is done because potentially enabling facts can still enable a new discovery at a future date. This lack of data about future events is handled analogously to a Kaplan–Meier curve to avoid biases due to right-censoring. One-edge networks are discarded.

In this study, each network node (A, B, C) is one of each a gene, a disease or a compound. Each edge is a relation (*e.g.*, a gene-disease relation) linked to a specific publication in the database MEDLINE. Data about relations came from The Comparative Toxicogenomics Database (CTD) (Davis et al., 2019), which was downloaded on May 4th, 2020. From this database, 1,603,976 unique relations between chemicals and genes were extracted; 34,830 relating genes and diseases and 218,868 relating chemicals and diseases. Additionally, co-occurrence data came from the MeSH and gene2pubmed databases. Chemical/drug and disease annotations were MeSH term annotations designated as “Major Topic” from the “Chemicals and Drugs” (D) and “Diseases” (C) branches, respectively, from the 2020 MeSH tree. Gene annotations came from the gene2pubmed database (Maglott et al., 2011) downloaded on August 20, 2020. These comprised 1,515,080 human gene annotations from 664,085 MEDLINE articles. MEDLINE data came from the 2020 MEDLINE/PubMed baseline. The reference date for each publication was the publication date (PubDate).

The citation distance was computed as the topological shortest-path distance between nodes in an undirected citation network in which the nodes were scientific publications recorded in MEDLINE and connections were citations between them (Rodriguez-Esteban, 2020; Rodriguez-Esteban, 2021). This citation distance differed from those described in previous work in that those typically involved directed connections (Botafogo, Rivlin & Shneiderman, 1992). The citation distance between any pair of publications was computed on citations existing at the time of publication of the latest article of the pair using bidirectional breadth-first search (BFS), which guarantees the finding of an optimal solution (Korf, 2003). Pairs of publications for which a path in the citation network could not be found were discarded from the analysis. A randomized version of the citation network was created by randomly swapping the nodes of the citation network, thus maintaining the network structure.

Citations came from the Open Citation Index repository (Peroni, Shotton & Vitali, 2017) and in particular from the March 23, 2020 update, which contained 721,655,465 citations between pairs of articles identified by a digital object identifier (DOI). DOI to PMID mappings were extracted from EBI’s PMID-PMCID-DOI dataset (Levchenko et al., 2018) downloaded on July 9, 2020, which contained 22,504,850 mappings between PMIDs and DOIs—thus covering 22,504,850 unique PMIDs in total. Using these mappings, 269,956,002 citations from the Open Citation Index were mapped from DOIs to PMIDs. As of July 2020, the fraction of publications covered by the Open Citation Index was 60% out of 51.1 million articles with references deposited with Crossref (https://i4oc.org/#about; checked on July 29, 2020).

The code used for this analysis is available at: https://github.com/raroes/scientific-silos.

## Results

**Figure 1:**
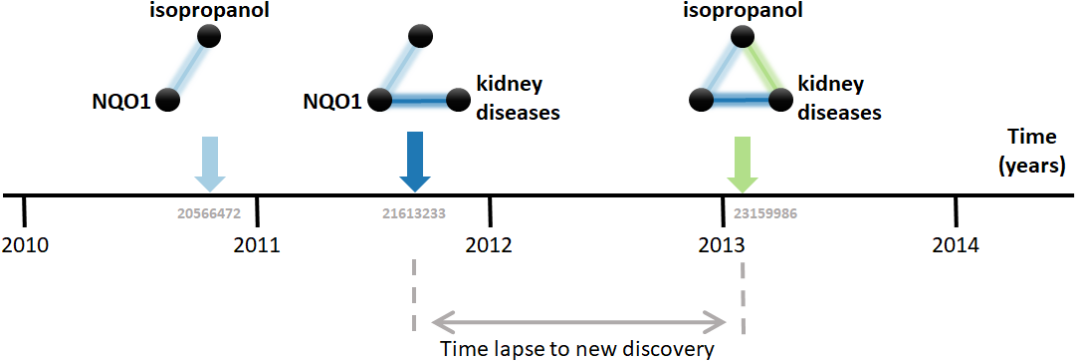
Connecting the dots. The three elements involved are the compound isopropanol, the gene NQO1 and kidney diseases. Each fact is a relation between two of these elements published in a MEDLINE article. For example, the relation between isopropanol and NQO1 was described in the article with PubMed ID 20566472 (Vandebriel et al., 2010). Data came from the Comparative Toxicogenomics Database (CTD).

Research on biomedical properties of compounds and genetic bases of disease is modeled here as a series of sequentially-built networks made of up to three nodes concerning each a gene, a compound and a disease. The nodes are connected by facts, which are molecular and medical relations published in the scientific literature. Central to this analysis is that two existing facts, *e.g.*, a gene-disease and a disease-compound relation, precede and, therefore, *enable* the posterior new discovery of another fact, *i.e.,* a gene-compound relation (Fig. 1). For example, the compound isopropanol leads to increased expression of the gene NQO1’s mRNA (Vandebriel et al., 2010). This, together with the fact that inhibition of NQO1 is linked to the amelioration of kidney diseases (Chen et al., 2011), enables a new discovery, namely the relation between isopropanol and kidney diseases (Brott et al., 2013). Using a comprehensive dataset containing thousands of such facts, this model can be employed to understand the dynamics of scientific discovery.

The first step in the analysis explored is to find all combinatorially-possible pairs of facts sharing an element in the dataset, such as all pairs of facts involving the gene NQO1. Together, these facts comprise all pairs of facts that can enable new discoveries. If a pair of these facts is followed by a new discovery, the time elapsed until that event is computed. *e.g.*, in Fig. 1, the time elapsed was between August 2011, when the second fact was published, and January 7, 2013, when the new discovery was published. This time elapsed is then used to estimate the pace at which scientists produce new discoveries from existing facts and, in the case studied here, to test its dependence on the “distance” between the publications in which the facts were published. The distance metric selected is the citation distance, which is a simple way to measure proximity in the scientific landscape (Rodriguez-Esteban, 2020). This distance is computed based on the citations existing at the time that the second fact is published. *e.g.*, in Fig. 1, the citation distance was 4 based on citation data from publications until August 2011, when the second fact was published. This distance would indicate how far apart the second publication was from the first within the scientific landscape at the moment of its publication, giving an indication of how separate the scientific milieus of these two publications were at the time in which they were both available to scientists.

**Figure 2:**
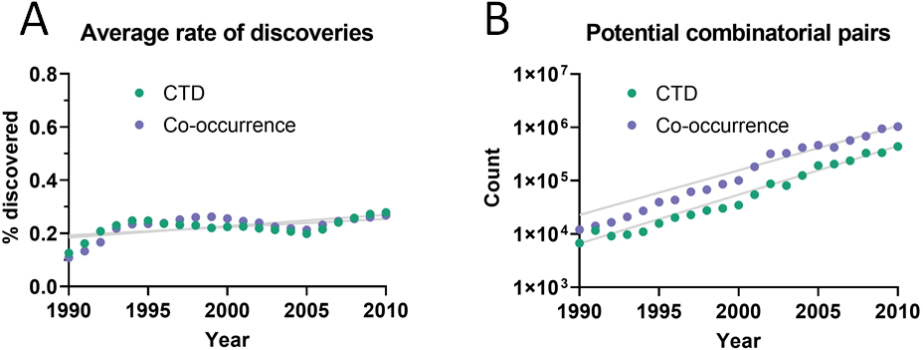
Pairs of facts enabling discoveries. (A) Rate of discoveries after 5 years averaged over a 10-year time window. *e.g.*, the earliest data point (1990) is an average for the period 1990-1999. Linear regressions were fitted to each curve. (B) Number of combinatorially-possible pairs of facts per year for each dataset. The year refers to the time when the second fact was published.

The dataset used initially for this analysis was the Comparative Toxicogenomics Database (CTD) (Davis et al., 2019), which contains manually-curated relations between compounds, diseases and genes from the literature. Out of all combinatorially-possible pairs of facts in the CTD (*n* = 6,261,706), only a small percentage (0.25%) was followed by new discoveries after 5 years. This percentage is here called “rate of discoveries,” and represents the percentage of all pairwise combinations of facts that precede a new discovery that combines them, such as in Fig. 1. The 5-year cutoff was chosen due to the reduced amount of data available for larger time windows (*e.g.*, 10 years). It can be seen in Fig. 2 that the rate of discoveries has changed only slightly over the decades despite power-law growth in the combinatorial possibilities.

**Figure 3:**
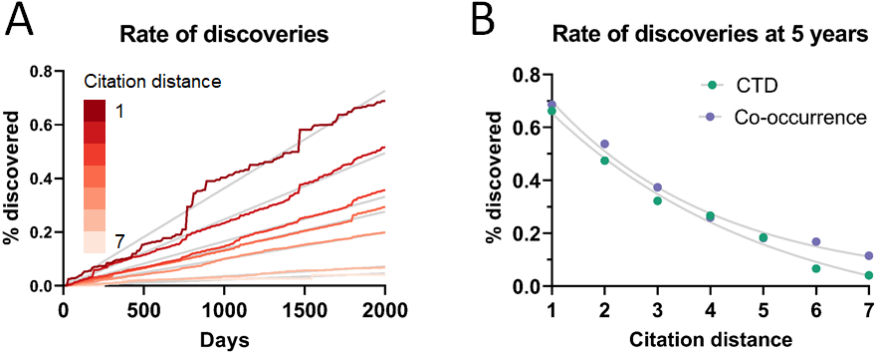
Rate of discoveries. Rate of discoveries (A) over time in CTD, and (B) after 5 years, based on citation distance. The rate of discoveries increased faster over time with smaller citation distance. Origin-intercept linear (A) and exponential (B) regressions were fitted to each curve.

As can be seen in Fig. 3A, the rate of discoveries increased linearly (goodness-of-fit s_y,x_ ranged from 0.03 for distance of 1 to 0.006 for distance of 7) over the years, as scientists had time to work with them. This rate, however, decreased with increasing citation distance, following an exponential decay (Fig. 3B shows the exponential decay at 5 years, which can also be seen for shorter time windows). For citation distance of 2, the rate of discoveries was, on average, 0.090% per year, while for citation distance of 5 it was an average of 0.036%. After 5 years, it was 2.6 times more likely that a new discovery would be made out of facts separated originally by a citation distance of 2 than out of facts separated by a citation distance of 5 (0.47% *vs.* 0.18%).

**Figure 4:**
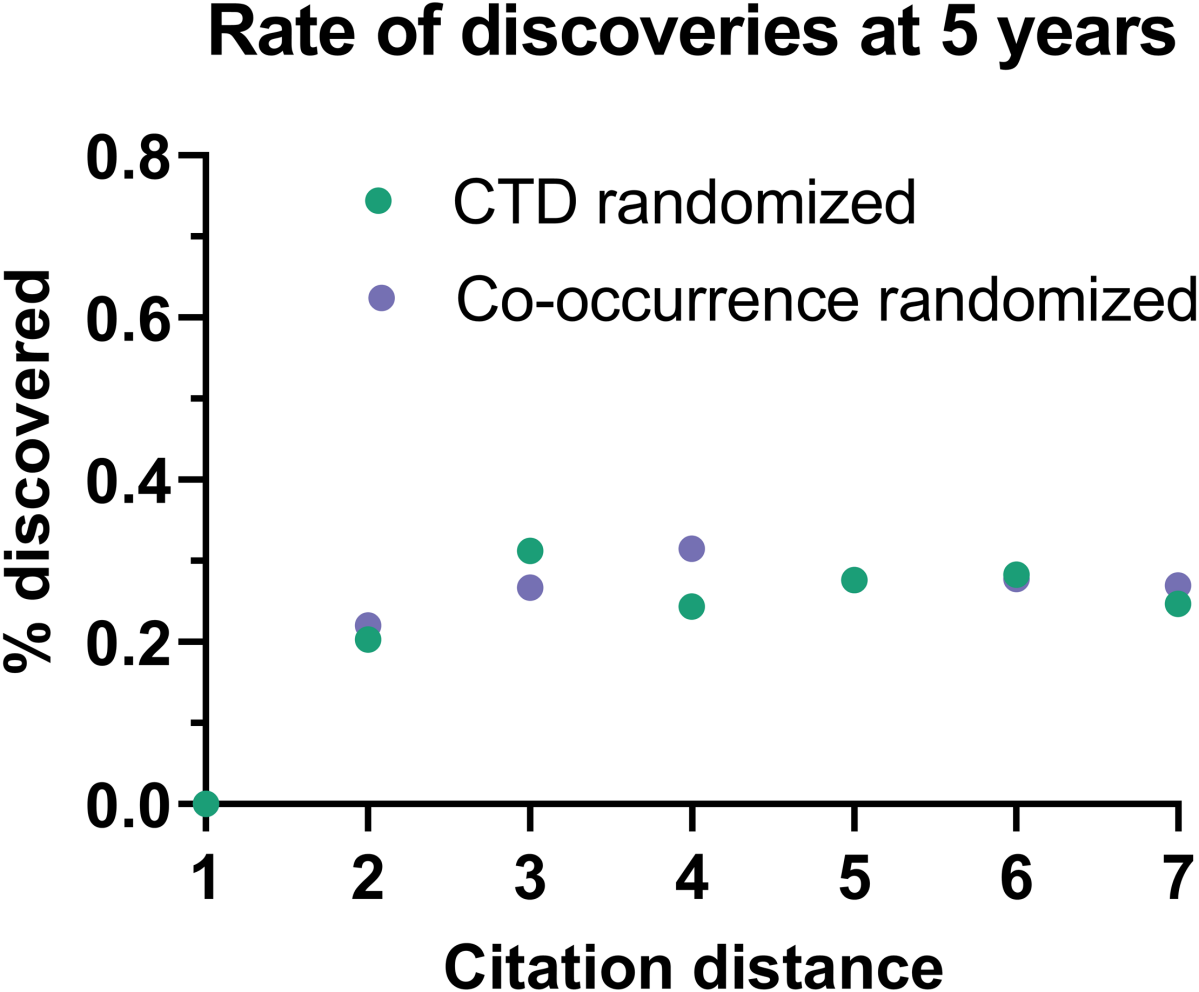
Rate of discoveries in a randomized citation network. Rate of discoveries after 5 years based on citation distance in a randomized citation network. Unlike in the non-randomized network, values did not follow an exponential decay based on citation distance.

This effect disappeared if all publications were randomly swapped within the citation network (Fig. 4). In this case, the rate of discoveries did not vary with citation distance, except for the case of distance equal to 1, due to data sparsity.

**Figure 5:**
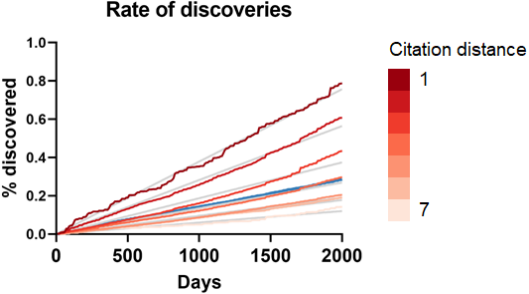
Rate of discoveries in the co-occurrence dataset. Rate of discoveries over time based on citation distance in the co-occurrence dataset. Origin-intercept linear regressions were fitted to each curve. Quadratic regressions were a better fit. The blue line represents the percentage for all citation distances.

To seek additional validation for these results, a similar analysis was performed with a different dataset based on co-occurrence of manual annotations of genes, diseases and chemicals/drugs of MEDLINE records. Co-occurrences have been considered suggestive of relations (Pavlopoulos et al., 2014) and have been used to discover new relations between drugs, genes and diseases (Frijters et al., 2010). The combinatorial space of all potentially enabling pairs of facts was three times larger (*n* = 17,040,304) in this case than for CTD but the overall outcome was similar (Fig. 5): The rate of discoveries was low (0.26%) 5 years after publication. The rate grew steadily with time, but at a different pace depending on the citation distance, following an exponential decay (Fig. 3). For facts separated by a citation distance of 2, the rate of discoveries increased, on average, 0.10% per year, while for a citation distance of 5, it was 0.035%. After 5 years, it was 3 times more likely that a new discovery was made out of facts published within a citation distance of 2 than out of facts within a citation distance of 5 (0.54% *vs.* 0.18%). This effect disappeared if publications were randomly swapped (Fig. 4). Similarly to the CTD case, the rate of discoveries did not vary with citation distance and was similar to the baseline, except for distance equal to 1 due to data sparsity.

**Figure 6:**
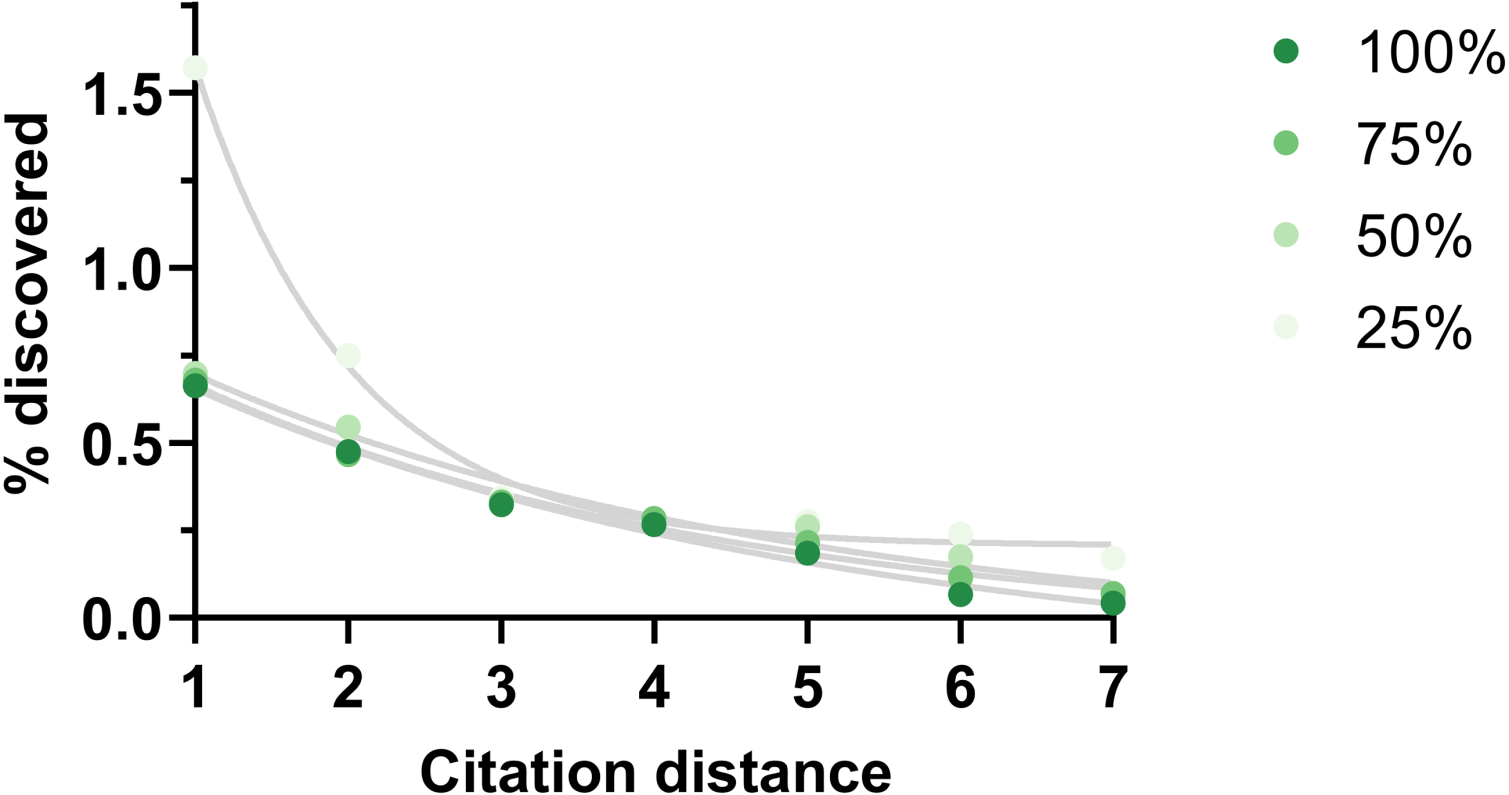
Rate of discoveries at 5 year after eliminating citations. Rate of discoveries after 5 years based on citation distance in a citation network with progressively less citations (100% = all citations available used, 75% = 75% of all citations available used, etc.). Exponential regressions were fitted to each curve. Data source was CTD.

One potential weakness of this analysis could be missing citation data. The effect of this shortcoming was examined by eliminating existing citations randomly. This reduction did not change the shape of the outcome except when it was large (75% reduction) (Fig. 6).

## Discussion

The fact that the analyses on both datasets led to similar outcomes lends some validation to the results. Both analyses show that, over time, scientists “connect” only a small percentage of existing facts about relations between compounds, genes and diseases. Thus, biomedical scientists appear to have a wide set of facts available from which they only end up publishing discoveries about a small subset of them, whether because of lack of resources, lack of interest, or because many combinations lead to negative results. Moreover, scientists steadily “accumulate” discoveries over the years but the rate of collective accumulation is higher when those discoveries concern facts that were originally closer within the citation network. This points towards a path-dependency in scientific discovery (Rzhetsky et al., 2015) in which originally closely-located discoveries drive the sequence of future discoveries rather than optimal unbiased choices.

As more facts are discovered, one may expect their potential combinations to grow quadratically, affecting, hypothetically, the propagation of scientific knowledge. However, there is a countervailing trend, which is that the scientific literature grows exponentially and it is able, therefore, to produce an increasingly larger number of discoveries. This analysis points to a somewhat stable relation between these two opposing forces. The overall percentage of facts that are being connected to form new discoveries has not changed much over the last decades and even increased slightly despite enormous growth in combinatorial possibilities (Fig. 2). If scientists were falling behind, we would expect to see a decrease. Additionally, the rate of accumulation of new discoveries (Figs. 3 and 5) appears generally stable and does not show signs of acceleration or deceleration over time (if only slight acceleration for co-occurrence data). Therefore, Swanson’s warning about “connection explosion” (Swanson, 2008) (“The literature of science cannot grow faster than the communities that produce it, but not so with connections. Implicit connections between subspecialties grow combinatorially. LBD [literature-based discovery] is challenged more by a connection explosion than by an information explosion.”) does not bear on this case, probably because scientists tend to confer a higher focus to a reduced set of drugs, diseases and genes (Yao et al., 2015; Haynes, Tomczak & Khatri, 2018; Stoeger et al., 2018; Rzhetsky et al., 2015), which would tend to limit combinatorial explosion.

The citation distance is only a rough estimate of scientific proximity between articles. One could expect that a more precise surrogate for scientific proximity could show an even stronger effect in the propagation speed. The citation distance was chosen for its simplicity. Alternatives such as measures of semantic similarity between articles could create cross-feedback between article annotations (*i.e.,* gene annotations) and the distance metric itself. The ABC method used assumes that the databases record every research finding and therefore it is possible to know the precedents of every new discovery. In practice, one can assume that there are precedents missing and, thus, that the speed by which new discoveries are based on existing knowledge is slower than it is described here. Moreover, lack of data would affect the number of combinatorial pairs more than the number of new discoveries and, therefore, the rate of discoveries should be, in fact, lower than here estimated. While we tested for robustness of predictions by eliminating citation data, we cannot be sure whether an increase in citation data would eventually have an important effect.

Finally, the simplicity of the ABC model misses important contextual information that would affect the computation of the speed of knowledge propagation. For example, if the results of an experiment were based on an animal model for a certain disease, one would expect that these results would propagate faster within the milieu of that disease rather than in other diseases, but that context is not captured in the model.

## Conclusions

This study shows how the appearance of new discoveries is more likely to be created from information that is closer within the scientific landscape. Reaching more often for facts that are closer could be a simple heuristic or a type of availability bias. That scientists may use heuristic biases, even if unconscious, to select their research goals should not be surprising, given the extraordinary growth of the scientific literature in most fields. However, this bias leads to a distortion of scientific progress and an opportunity for those who may venture further away from their scientific milieu with the aid of modern tools (Krenn & Zeilinger, 2020; Whalen et al., 2016; Raymond, 2019). If scientists increased the reach of their search scopes, one would expect the propagation speed of information to increase. That a phenomenon such as siloization could derive from a slowdown in information propagation in the scientific network is ultimately an emerging property of scientific organization and self-organization with cognitive, social and technological aspects.

## Additional Information and Declarations

### Data Availability

The following information was supplied regarding data availability: All code used in this article is available at GitHub: https://github.com/raroes/scientific-silos.
